# Notes on Surgery

**Published:** 1875-08

**Authors:** W. W. Miner


### MEDICAL NOTES.

ART. I.—*Notes on Surgery.* By W. W. MINER, M. D.

1. Least Sacrifice of Parts as a Principle of Surgical Practice. By THOMAS BRYANT, F. R. C. S. (*London Lancet*, April and May, 1875.)

2. Resection of Metatarsus, Anterior Tarsus, and parts of Astragalus and Os Calcis. Recovery with Useful Foot. By P. S. CONNOR, M. D. (*American Journal Medical Sciences*, July, 1875.)

3. Recent Improvements in the Details of Antiseptic Surgery. By JOSEPH LISTER, F. R. C. S. (*London Lancet*, June, July, Aug., 1875, et seq.)

4. Clinical Results of Lister's Treatment of Wounds, and substitution of Salicylic Acid for Carbolic Acid. By Prof. C. THIERSCH, Leipsic. (*Volkmann's Collection of Clinical Lectures.* Nos. 84 and 85.)

5. Comparative Antiseptic Properties of Salicylic and Benzoic Acids. By Prof. E. SALKOWSKI. (*Berliner Klinischer Wochenschrift*, May 31st, 1875.

I. A recent article in the *Lancet* which is indicative of the remarkable results obtained in this country and abroad, in cases of injury or disease affecting the bones and joints of the tarsus, is one by Thomas Bryant, Surgeon to Guy's Hospital, and author of Bryants Practice of Surgery. He gives the history of several of his own cases in which removal of bone was practiced, with good results. Beside removal of the scaphoid, internal cuneiform, and base of metacarpal bones, he gives cases in which he removed portions of dead bone which had been the articular surface of the os calcis, also the lower half of the astragalus, with good recovery.

Mr. Bryant advocates free incisions into a diseased joint and removal of any diseased bone that may be the cause of trouble, without excising the joint surfaces. As soon as it becomes evident, from the formation of abscesses or sinuses, that suppurative disease of a joint has taken place, he makes a free incision, removes any sequestra or diseased bone that my appear, and leaves the parts in a condition for a natural process of repair. He says:

"I feel more convinced year by year that disorganized joints are more amenable to direct local surgical treatment than is generally supposed, and that free incisions into joints, and the removal of necrosed bone from joints, are both excellent and successful operations.

A free cut into a suppurating or disorganized joint whether associated or not with bone disease, is rarely followed by any other than a good result. When the suppurating process is due to synovial disease, a recovery without further surgical interference may be looked for. When due to local necrosis, the incision helps nature towards the recovery of the cases by expediting the process of exfoliation, and the subsequent removal of the bone by either natural processes or some surgical proceeding, and in still more severe cases, the incision gives relief, and in no way tends toward inducing any increase in the mischief."

After giving the histories of some twenty or thirty cases, more or less illustrative of this plan, he concludes:

"I trust this series of cases is enough to demonstrate with sufficient clearness, the value of the practice I am now inculcating, and to show that in a large number of cases of disease of the joints, a cure may be secured by a simple incision into the affected joints and the removal of the necrosed bone. This series includes examples of disease of the shoulder, elbow, hip, knee, ankle, and great toe joints, and I do not think I should be far wrong if I were to express my belief that in many of these cases, if not in all, many surgeons, more particularly those who are advocates for excision—would have excised the joints and some few would have amputated. I am not here however to condemn their practice, for their results might have been good; but whatever they might have been, they would have been secured by severe operative measures, and consequently by dangerous risks, whereas in the treatment I am now advocating, the surgical proceedings are simple, and are attended with a minimum of danger. The success of the practice I have recorded was also great—indeed, it has been so good, that I am induced to express my belief that almost all operative measures upon diseased joints, should be in a measure exploratory, in order that necrosed bone if present may be taken away, and the case then left to natural processes; with Esmark's bandage this practice is rendered more simple and is consequently likely to be more satisfactory."

II. Prof. P. S. Connor, M. D., of Cincinnati, gives the history of a case of disease of the tarsus, following injury, in which he removed all the bones of the foot between the astragalus and calcis posteriorly and the phalanges in front. He presents a cut of the extremity as it appears after recovery, and says it forms a very excellent basis of support, and that the ordinary movements of walking are well performed. Accompanying his case, he presents a tabular view of thirty-five recorded cases, with name of operator, parts removed, results, where recorded and observations thereon.

III. Professor Lister is still very enthusiastic in his researches for the advancement of antiseptic surgery. Besides the carbolic lotion, varying from one to five per cent. in strength, he uses the watery solution in the form of atomized spray, and prefers for this purpose, and ordinarily, to have the solution of two and one-half per cent strength ; one per cent. being that formerly recommended in his address before the British Medical Association in 1871. A favorite method of dressing with him now is that of antiseptic guaze. This is common thin gauze which has been permeated with a mixture containing one part of carbolic acid, five of resin, and seven of paraffine. Eight layers of this antiseptic gauze are successively applied to an open wound, and outside of this is carefully wrapped a piece of cloth which has been rendered impervious by a coating of caoutchouc. It is intended that such dressing should remain undisturbed for from two days to a week. Carbolized oil he generally uses of a five per eent. strength. Chloride of zinc he has found very efficient to prevent extension of suppurative processes. A single application of it, forty grains to the ounce is used. Boracic acid he has found a useful antiseptic remedy. Boracic lint is prepared by dipping lint in a saturated watery solution of boracic acid heated to near the boiling point, and then allowing it to dry. It is used as a dressing for indolent ulcers; is dipped again when used, in a cold watery solution of the same and applied after a vivifying application of chloride of zinc has first been made. This acid is much more soluble in hot than in cold water; near the boiling point, water takes up one-third its weight, at ordinary temperatures one twenty-sixth its weight of the acid. He uses boracic applications in skin grafting, burns, fresh wounds, etc. An ointment of boracic acid is sometimes found advantageous, and he advises the following mode of preparation:—Boracic acid finely levigated, and white wax, each, one part; paraffine and almond oil, each, two parts; mix these thoroughly by heat and trituration.

Accounts of the visit Professor Lister is making through the German cities, where he has been enthusiastically welcomed, represent him as saying that he has found salicylic acid inferior in value to carbolic acid. This statement was made at Leipsic, the home of Professor Thiersch, who is an interested observer of the action of salicylic acid, and who has published important articles upon this new agent, which is now having extensive trial among the profession.

V. Professor Salkowski, of Berlin, has been making experiments with various disinfectants to determine their relative value, and as between salicylic and benzoic acids, he reports results markedly in favor of benzoic acid. Salicylic acid in concentrated aqueous solutions will retard putrefaction, but not prevent it. It is not a deodorizer in other sense than that of hindering putrefactive change. Salicylic, as well also benzoic acid, which he says has stronger antiseptic properties, are alike unfitted for internal use, because they are converted in the blood into salts of soda, whereas phenol and allied substances will pass unchanged through the system. His statements in this respect are opposed to those of Kolbe's, and time is required to determine the value of salicylic acid which has many advocates now among experimenters.

